# Improved Feature Matching for Mobile Devices with IMU

**DOI:** 10.3390/s16081243

**Published:** 2016-08-05

**Authors:** Andrea Masiero, Antonio Vettore

**Affiliations:** CIRGEO (Interdepartmental Research Center of Geomatics), University of Padova, via dell’Università 16, 35020 Legnaro (PD), Italy; antonio.vettore@unipd.it

**Keywords:** 3D reconstruction, photogrammetry, feature matching, inertial navigation system, smartphones

## Abstract

Thanks to the recent diffusion of low-cost high-resolution digital cameras and to the development of mostly automated procedures for image-based 3D reconstruction, the popularity of photogrammetry for environment surveys is constantly increasing in the last years. Automatic feature matching is an important step in order to successfully complete the photogrammetric 3D reconstruction: this step is the fundamental basis for the subsequent estimation of the geometry of the scene. This paper reconsiders the feature matching problem when dealing with smart mobile devices (e.g., when using the standard camera embedded in a smartphone as imaging sensor). More specifically, this paper aims at exploiting the information on camera movements provided by the inertial navigation system (INS) in order to make the feature matching step more robust and, possibly, computationally more efficient. First, a revised version of the affine scale-invariant feature transform (ASIFT) is considered: this version reduces the computational complexity of the original ASIFT, while still ensuring an increase of correct feature matches with respect to the SIFT. Furthermore, a new two-step procedure for the estimation of the essential matrix *E* (and the camera pose) is proposed in order to increase its estimation robustness and computational efficiency.

## 1. Introduction

3D reconstruction techniques have been widely used in the last decades in order to produce digital models of the environment [1,2]. Climate change [3], the recent worldwide increase of natural hazards, the need for cost-effective solutions for continuously monitoring risky environments and the growing request for high resolution reconstructions [4] motivate the constant great interest of the research community on the search for improvements of 3D reconstruction and modelling techniques.

Terrestrial and airborne laser scanning methods can be considered as state-of-the-art for high resolution surveys in the last dozen years [5]. However, laser scanning instrumentation is usually quite expensive and its use is mostly restricted to high qualified personnel. On the contrary, photogrammetric methods are typically quite inexpensive, fast and more prone to the use of unqualified operators. Furthermore, the commercialization of low-cost high resolution cameras and the recent development of mostly automated procedures allowing for obtaining dense 3D photogrammetric reconstructions are making photogrammetry more and more attractive for cost-effective surveys. A significant contribution to this purpose is provided by the spread of new smart mobile devices (e.g., smartphones, tablets and action cameras, which can allow to anyone to acquire high resolution images to be used for photogrammetric reconstruction, as also shown in recent works on 3D reconstructions based on smartphones [6,7,8,9,10,11,12]) and vehicles for data acquisition (e.g., unmanned aerial vehicles (UAVs)), which can be low-cost solutions to monitor areas difficult to reach by terrestrial acquisition systems [13]). The success of this combination of factors can be seen in the recent flourishing research production on photogrammetric surveys [14,15,16].

As mentioned above, photogrammetric reconstruction has the potential for further increasing its importance in surveying applications, in particular if high resolution, robustness and accuracy can be improved to a level similar to that of laser scanning methods: the goal of this paper is that of partially modifying the feature matching procedure in order to improve the performance for what concerns the criterions listed above.

Once images of the scene of interest have been acquired, the workflow procedure of recent photogrammetric methods can be summarized as follows:

1. Feature extraction. Characteristic points of the images are extracted and the corresponding descriptors are computed [17,18,19,20].
2. Feature matching and geometry estimation (Structure from Motion (SfM)). Feature matches are computed in couple of images. These correspondences are used in order to estimate the geometry of the scene [21,22,23,24,25,26] (camera poses and point positions). Geometric constraints are exploited to detect wrong feature matches, if any.
3. Dense reconstruction. Starting from the previously estimated geometry, pixel-level matches are used to produce a dense point cloud [27,28,29] (triangulation by means of matched point positions [30,31,32]).

The minimum requirement of the above procedure is the availability of a sufficient number of (partially overlapping) images. Nevertheless, the recent development of low-cost MEMS sensors [33] (which nowadays are embedded in most of the new mobile devices (e.g., smartphones and tablets)) opens the possibility of integrating more information in the reconstruction procedure, in order to make it more robust [34,35].

To this aim, several works in the literature already considered the use of camera orientation information: in [36], orientation is estimated from vanishing points in urban environments, and (assuming camera interior parameters as known) this information is used to remove 3D rotation effects. In [34], information about gravity direction is effectively employed in order to improve matching in particular for (close to) vertical or (close to) horizontal surfaces). Observability analysis in a vision–aided Inertial Navigation System (INS) is provided in [7]. Information provided by the inertial measurement unit (IMU) is also used in [35] to estimate the essential matrix by using two point correspondences.

This paper proposes the following changes to the second step of the reconstruction procedure summarized above:

- Increase the number (and/or the percentage) of correct feature matches between two camera views (in particular for wide changes of the camera point of view, Section 2). A number of feature descriptors have been proposed in the literature [17,18,19,20]. Despite the fact that they are typically designed to deal with certain image transformations (e.g., scale changes), sometimes they are not effective in dealing with generic changes of the camera point of view. The Affine Scale-Invariant Feature Transform (ASIFT, or affine SIFT [37,38]) has been recently introduced to improve the SIFT performance in this case. The method presented in Section 2 can be considered as a revision of the ASIFT when prior information from the device orientation is available. The goal considered here is similar to that in [34,36]; however, in [36], device orientation is provided by vanishing points (whereas here, orientation is provided by the IMU) and the method in [36] is designed for buildings. Instead, [34] exploits gravity direction information, whereas relative orientation information is used in Section 2: interestingly, the method considered in [34] can be integrated in the procedure of Section 2 (this aspect will be the subject of future investigation). Since the interest of increasing the number of correct feature matches. Since the net effect of the method presented in Section 2 (increasing the number (and/or percentage) of correct feature matches) is of particular interest when dealing with quite large changes of the point of view, it is also related to the wide–baseline stereo (WBS) matching problem: differently from standard approaches for WBS [39,40,41,42], here the problem is addressed by adding information provided by the IMU about the camera orientation.
- Feature matching with geometry constraints: assuming interior camera parameters as known, then an estimate of the essential matrix (and hence the relative camera pose) between two camera views is computed by means of two feature correspondences (this two-point procedure is the same proposed in [35], with only minor changes). This is employed in a two-step RANdom SAmple Consensus (RANSAC, [43]) procedure in order to make the estimation more robust (Section 3): the goal is that of removing wrong feature matches and correctly estimating the geometry of the scene (i.e., the essential matrix). It is well-known that the number of iterations of the RANSAC algorithm (necessary in order to obtain a good estimate with the desired probability) is often underestimated because only certain subsets of the inliers allow for obtaining a correct estimation [44]. Similarly to the locally optimized RANSAC [44], the procedure presented in Section 2 aims at compensating for this issue by preselecting a candidate inlier subset where the probability of drawing a correct feature match is higher than in the original set of feature matches. However, differently from [44], this preliminary step is done by exploiting information provided by the sensors embedded in the device.

## 2. Similarity Based Feature Matching

Most of the smart mobile devices sold in the last years are provided with an IMU and an INS: thanks to the presence of the INS, even in environments challenging for the Global Navigation Satellite System (GNSS) positioning system (e.g., indoors, close to high buildings or mountains), an estimate of its movements is available to the device [45,46,47,48].

Hereafter, the information provided by the INS is assumed to be available to the 3D reconstruction system. To be more specific, just an estimate of the device orientation is assumed to be available (e.g., computed by processing the IMU measurements: in this work (at least) a three-axis accelerometer and three-axis magnetometer are assumed to be embedded in the device).

Despite several feature descriptors having been recently proposed in the literature, the methods presented in this work are developed and validated by using the SIFT. This choice is motivated by the widespread use of the SIFT, and by its very good quality performance (in terms of percent of correct feature matches). Nevertheless, this choice should not be considered as a strict requirement for the proposed methods: indeed, a similar approach can be deployed for other feature descriptors as well.

A feature point is a specific part of the image whose position in the image can be accurately determined and that, thanks to its appearance characteristics, can be distinguished from other image points. Thanks to this property, features are used in order to match corresponding points in different images [17]. Since images to be compared are typically taken from different positions (and sometimes in different illumination conditions), the appearance of a spatial point can change in different images: specific feature descriptors are computed in order to be (partially) invariant to such variations of the appearance of the same spatial point in different images.

**Figure 1:**
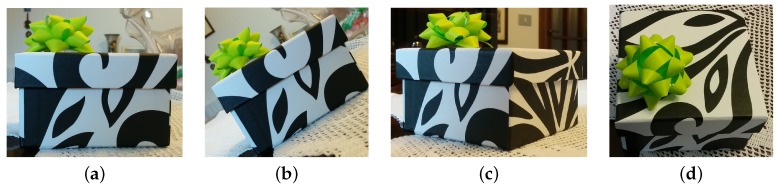
Examples of image views obtained by rotating the camera along three (approximately) orthogonal axes. (**a**) original camera pose; (**b**) rotating along the optical axis of the original camera pose; (**c**) rotating the camera towards the right of the object; and (**d**) rotating the camera towards the top of the object.

As is widely known, SIFT descriptors allow for obtaining reliable matching between features in two images when the images are related by certain transformations, e.g., different scalings, illumination and rotations on the image plane (see Figure 1b). However, matching issues can occur when in the presence of different changes of the point of view between the two images (e.g., rotations along the other two axes as in Figure 1c,d). As shown in [37,38], in this case, local changes between features in the two images can be usually well represented by means of affine transformations.

In order to make the SIFT descriptor robust even to these kind of transformations, Morel and Yu [37,38] proposed simulating N=32 versions of each of the original images, where each version simulates the appearance of the corresponding image after applying a specific affine transformation. The *N* affine transformations can be associated to different values of the rotation angles, i.e., they represent a discretization of the set of possible rotations that provide variant results to the SIFT descriptors (combinations of rotations as in Figure 1c,d). Then, feature points are extracted in all the *N* versions of the images. Thus, when searching for feature matching between image $I_{1}$ and $I_{2}$, all the corresponding SIFT descriptors are matched: all the possible combinations of matches are checked, i.e., the features extracted in each of the *N* versions of $I_{1}$ are matched (when possible, according to the standard SIFT matching procedure, [17]) with the features extracted in each version of $I_{2}$. In accordance with the type of transformations applied to each image, this procedure is named affine SIFT (ASIFT).

Despite the face that the computational complexity of ASIFT is clearly larger with respect to that of the SIFT, it can be reduced by considering multi-resolution representations of the images: as shown by [37,38], the computational load required to compute the ASIFT descriptors can be reduced to approximately 2.25 times that of the SIFT. However, even in this case (assuming that each version of the same image contains approximately the same number of features), the time for matching the features is approximately $N^{2}$ times that of the original SIFT.

The goal of this section is that of proposing an alternative feature matching method that similarly to the ASIFT allows for increasing the robustness of the feature matching step to all types of rotations (e.g., Figure 1c,d), while significantly reducing the number of comparisons to be done with respect to the ASIFT. To be more specific, the method developed here can be considered an extension of the ASIFT when information of the pose of the camera is available (e.g., provided by the IMU measurements).

Since positioning algorithms allow for providing real time positions and orientations of the device (e.g., exploiting the IMU measurements), the proper rigid transformation between each couple of camera poses while acquiring the images is approximately known. Consider the feature matches between the images acquired by camera 1 and 2: the rationale of this work is that of exploiting the pose information provided by the IMU in order to simulate how the object seen by camera 2 is viewed by camera 1, and vice versa. This task is similar to that of standard rectification: given the relative orientation between two images and assuming a planar scene, it is possible to create an image of a virtual camera that allows for significantly removing the orientation ambiguity. However, in order to accurately reproduce the appearance of the features from other points of view, in this work, the scene is not assumed to be planar, and local orientations (of the scene surface in the neighborhood of the features) are considered instead. Actually, such orientation is not known a priori, hence the reproduction is done conditioning on the local surface value, for different values of such orientation.

As in standard rectification, a camera intrinsic matrix should be known to compute accurate rectified images. However, in order to check the possibility of using the proposed method even in uncalibrated scenarios, the results shown in Section 4.1 are obtained without calibrating the camera: instead, the intrinsic matrix is approximated as in [49,50] (and exploiting information provided by the device operative system on the camera geometric characteristics).

In practice, the space of the local surface orientations is sampled in *N* orientations, and the appearance of each image is simulated along such orientations (in our simulations, N=25 orientations, distributed on the semi-sphere centered in the original observing direction of the camera). In accordance with the above considerations, these orientations are samples of the possible orientations of the local surface of the object represented in the image.

Similarly to the ASIFT, feature locations and descriptors are computed for each of the *N* versions of the images. Then, differently from ASIFT, feature matching between features from two images is performed *conditioning on the surface orientation* of the object of interest represented in the images. Since the object surface orientation is unknown, all the *N* simulated orientations have to be considered. However the features in the $k_{1}$-th simulated orientation of $I_{1}$ are compared only with those of the $k_{2}$-th simulated version of $I_{2}$, where $k_{2}$ is chosen in order to make the two orientations as close as possible (since the space of orientations has been discretized, whereas camera orientation and object shape do not have any restriction, typically the orientations in $I_{1}$ and $I_{2}$ cannot be equal).

**Figure 2:**
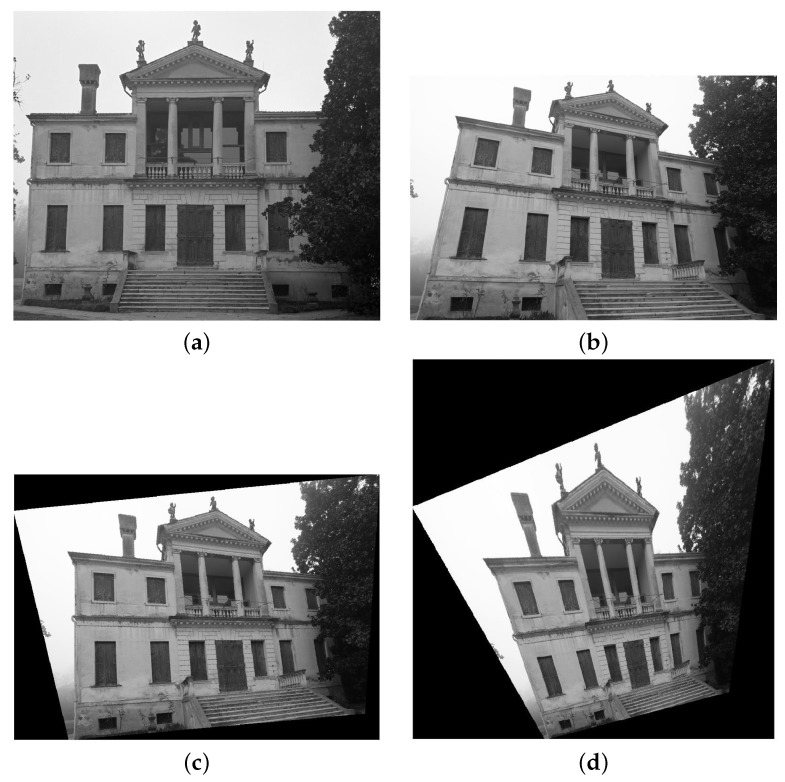
Example of image transformation to improve feature matching when dealing with generic change of the point of view. (**a**) image 1; (**b**) image 2; (**c**) image 1 transformed in order to improve feature matching with image 2; and (**d**) image 1 after applying a transformation not properly related with image 2.

For instance, consider the images of Figure 2a,b, where the façade of a villa is represented. In this case, the surface of the observed object is clearly characterized by a dominant orientation: the façade of the villa is approximately parallel to the image plane of Figure 2a, hence conditioning on this orientation, the transformation of Figure 2b will be closer to that corresponding to the real change of observation orientation between the two images (represented by the matrix *R*, which is approximately known thanks to the IMU measurements), as shown in Figure 2c. It is worth noticing that the change of orientation has not been completely compensated in Figure 2c: this is due to the noisy measurement of the IMU, to the use of an uncalibrated camera, and to the discretization of the space of orientations (only *N* orientations are simulated). Instead, associating the same change of orientation *R* to image in Figure 2a but assuming the orientation of the represented object vertically tilted by 30 degrees, then the result is that of Figure 2d (which is clearly definitely worse than that of Figure 2c).

Since, in the matching procedure, *N* hypothetical orientations are considered for the local surface of the observed object, then the procedure is able to deal also with objects with shapes less regular than that of Figure 2.

It is worth noticing that the the choice of the $k_{2}$-th orientation (of the second image) closest to the $k_{1}$-th of the first image has been allowed by means of the information provided by the navigation sensors: this way, the number of comparisons to be done (between different versions of the original images) can be reduced from $N^{2}$ (in the ASIFT) to *N* (in the proposed procedure), hence significantly reducing the computational time required at this step. This information can also be used to obtain more robust feature matching: the approximate knowledge of the different camera poses and of the intrinsic camera parameters (e.g., the values of focal length and pixel size can be approximately obtained by proper queries to the operative system of the device) allow for computing an approximate fundamental matrix (or essential matrix, depending on if the camera interior parameters are known or not), hence using an (approximate) epipolar constraint in order to discard wrong feature matches at an early stage of the matching procedure.

Since only a relatively small number of orientations *N* is considered for each image, then, despite the $k_{2}$-th orientation of the second image having been chosen as the closest to the $k_{1}$-th of the first image, such orientations will typically differ due to the discretization of the space of orientations. Instead of using only the closest orientation, the method presented above can be generalized by considering the *h*-th closest orientations (in the second image) to the $k_{1}$-th of the first image (for each value of $k_{1}$). This generalization implies a linear increase of the computational burden with respect to *h*. If h=N, then the computational burden becomes similar to that of the original ASIFT.

## 3. Feature Matching with Geometry Constraints: Estimation of the Essential Matrix

The rationale of this section is that of exploiting IMU measurements in order to increase the robustness and computational efficiency of automatic procedures for estimating the feature correspondences between two camera views while taking into account of the (estimated) geometry of the scene (e.g., the essential matrix).

Since the goal of the considered system is that of providing accurate photogrammetric reconstructions, in order to obtain accurate reconstructions, the camera hereafter is assumed to be calibrated (i.e., interior calibration camera parameters are assumed to be known [51,52,53,54,55,56,57,58]): automatic estimation of the pairwise image matching (feature-point correspondence determination and RANSAC-like outlier detection) and, consequently, of the essential matrix (i.e., of the geometry of the scene) is a fundamental step in the reconstruction procedure, and can even be used combined with traditional rigorous photogrammetric methods in order to obtain accurate 3D modelling [58].

Once incorrect feature matches have been removed and the essential matrix has been estimated, then the latter allows the estimation of the relative camera poses [21] (e.g., to compute the position and orientation of a camera with respect to another).

Camera interior parameters are assumed to be known, hence without loss of generalization measurements can be assumed to be undistorted and normalized, i.e., distortion is negligible or has already been compensated and the camera measurement process can be modeled as follows:

(1) $$p_{ij}=K_{j}\;\left[R_{j}\;|\;-R_{j}t_{j}\right]\;M_{i}$$

where the interior parameter matrix $K_{j}$ of camera *j* (for all the considered values of *j*) is the identity, and $R_{j}$, $t_{j}$ are the camera orientation matrix and the position, respectively.

Independently of the specific considered feature descriptor and appearance based matching criterion (e.g., [17,59] or as in Section 2), similarity appearance matching usually provides a certain number of wrong features matches. This motivates the use of an outlier rejection procedure in order to remove wrong feature matches from the camera pose estimation procedure. This is usually achieved by using a RANSAC estimation procedure and checking the geometry consistency of the matched feature points by means of the estimated essential matrix.

In each iteration of the RANSAC procedure, the computation of the current estimate of the essential matrix is usually done by means of a minimal set of parameters, i.e., by using either the five-point or the linear eight-point algorithms [60,61,62]. The possibility of computing the essential matrix with less points clearly leads to the advantage of reducing the number of necessary RANSAC iterations for the estimation, or, alternatively, to make the estimation more robust.

Motivated by the above considerations, first, this section resumes a method for exploiting IMU measurements in order to allow a two-point estimation of the essential matrix: this procedure, up to a few minor changes, is the same proposed in [35] (the reader is referred to [35] for a comparison on the use of such a two-point procedure in RANSAC and Hough transform based estimations). Then, a two-step algorithm is proposed in order to obtain an accurate estimate of the essential matrix: the two-point algorithm is used to obtain quick estimates of the essential matrix, and then the classical five-point algorithm is applied only to the inlier matches of the previously obtained estimate in order to increase the accuracy of the estimation procedure (but at a lower computational cost with respect to a direct use of the five-point algorithm).

### 3.1. Two-Point Estimation of the Essential Matrix

The aim of this subsection is that of showing a procedure for estimating the essential matrix (summarizing the geometry between camera 1 and 2) by using the correspondence of two features in the two image planes (the reader is referred to [35] for a detailed description of this procedure and of its comparison with an estimation based on the Hough transform). Without loss of generality, let the projection matrices of camera 1 and 2 be as follows:

(2) $$P_{1}=\left[I\;|\;0\right]\;\;,\;\;\left[R_{2}\;|\;-R_{2}t_{2}\right]=\left[R_{2}\;|\;t_{2}^{\prime }\right]$$

where $t_{2}^{\prime }=-R_{2}t_{2}$.

The essential matrix $E_{21}$ relating the two considered camera views is defined by the following equation:

(3) $$E_{21}={\left[t_{2}^{\prime }\right]}_{\times }R_{2}$$

where ${\left[\cdot \right]}_{\times }$ indicates the matrix representation of the cross product (i.e., ${\left[a\right]}_{\times }b=a\times b$, for each choice of the two vectors *a* and *b*):

(4) $${\left[t_{2}^{\prime }\right]}_{\times }=\left[\begin{array}{ccc} 0 & -t_{2}^{\prime }\left(3\right) & t_{2}^{\prime }\left(2\right)\\ t_{2}^{\prime }\left(3\right) & 0 & -t_{2}^{\prime }\left(1\right)\\ -t_{2}^{\prime }\left(2\right) & t_{2}^{\prime }\left(1\right) & 0 \end{array}\right]$$

where $t_{2}^{\prime }={\left[t_{2}^{\prime }\left(1\right)\;\;t_{2}^{\prime }\left(2\right)\;\;t_{2}^{\prime }\left(3\right)\right]}^{⊤}$.

According to the definition of essential matrix, the following equation relates two corresponding measured locations (in undistorted normalized coordinates), $p_{i1}$ and $p_{i2}$, of the same feature *i* in two camera views:

(5) $$p_{i2}^{⊤}E_{21}p_{i1}\approx 0$$

where, in practical applications, due to measurement noise and to estimation errors, the left side of the above equation is only approximately equal to zero.

Then, the following can be obtained by substituting Equation (3) in the above equation and properly re-arranging the terms:

(6) $$\left[\begin{array}{ccc} q_{i1}\left(2\right)p_{i2}\left(3\right)-q_{i1}\left(3\right)p_{i2}\left(2\right) & q_{i1}\left(3\right)p_{i2}\left(1\right)-q_{i1}\left(1\right)p_{i2}\left(3\right) & q_{i1}\left(1\right)p_{i2}\left(2\right)-q_{i1}\left(2\right)p_{i2}\left(1\right) \end{array}\right]t_{2}^{\prime }\approx 0$$

where $q_{i1}=R_{2}p_{i1}$, whereas $q_{i1}\left(h\right)$ and $p_{i1}\left(h\right)$ indicate the *h*-th component in the vectors $q_{i1}$ and $p_{i1}$, respectively.

Since projective measurements provide information up to a scale factor, $t_{2}^{\prime }$ can be assumed to be a unit vector: hence, $t_{2}^{\prime }$ can be estimated by using two feature point correspondences by using (6): let (6) be re-written as $At_{2}^{\prime }\approx 0$, and $A=[A_{12}\;|\;A_{3}]$, where $A_{12}$ and $A_{3}$ are the matrices formed by the first two columns and the third column of *A*, respectively. Then, three cases can be distinguished:

- if $t_{2}^{\prime }\left(3\right)=0$, then $\hat{x}=\arg\min_{x}={∥A_{12}x∥}^{2}$, and $t_{2,1}^{\prime }={\left[{\hat{x}}^{⊤}\;0\right]}^{⊤}$;
- $t_{2}^{\prime }\left(3\right)>0$, then $t_{2,2}^{\prime }={\left[{\left(A_{12}^{†}\right)}^{⊤}\;1\right]}^{⊤}$;
- $t_{2}^{\prime }\left(3\right)<0$, then $t_{2,3}^{\prime }={\left[{\left(A_{12}^{†}\right)}^{⊤}\;-1\right]}^{⊤}$.

The three estimates $\left\{t_{2,\cdot }^{\prime }\right\}$ above have to be normalized, and then the one leading to the least square error is selected as the estimate of $t_{2}^{\prime }$.

As shown in Algorithm 1, the two-point algorithm can be included in a RANSAC procedure for determining wrong/correct feature matches and for the estimation of the geometry of a scene.
**Algorithm 1:** Essential matrix RANSAC estimation with the two-point algorithm
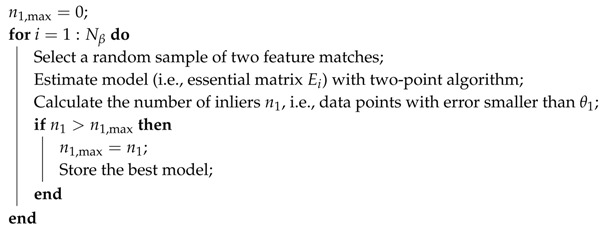


In Algorithm 1, $n_{1}$ and $n_{1,\max}$ are the number of inliers of model $E_{i}$ and of the best model at the current RANSAC iteration, respectively.

The number of iterations $N_{\beta }$ of the RANSAC algorithm is usually determined taking into account the probability of correct feature matching (i.e., the probability, assumed to be known a priori, that a feature match is correct, e.g., β=0.7), and of the probability of computing a wrong estimate of the essential matrix (e.g., $\epsilon =10^{-4}$). $N_{\beta }$ is computed assuming that every time that a correct couple of feature matches are drawn, then it is possible to obtain a correct estimate of the essential matrix (i.e., an estimate such that (almost all) the correct feature matches can be determined). Based on such an assumption, $N_{\beta }$ is computed as follows:

(7) $$N_{\beta }=\frac{\log\epsilon }{\log(1-\beta^{2})}$$

The above equation can be easily generalized for a generic number of points *n* required for the estimation (e.g., in the five-point algorithm n=5, obviously):

(8) $$N_{\beta }=\frac{\log\epsilon }{\log(1-\beta^{n})}$$

Finally, a couple of feature points are considered as inliers if

(9) $$\frac{{\left(p_{i2}^{⊤}E_{21}p_{i1}\right)}^{2}}{p_{i1}^{⊤}E_{21}^{⊤}E_{21}p_{i1}+p_{i2}^{⊤}E_{21}E_{21}^{⊤}p_{i2}}\leq \theta$$

where *θ* is a predefined threshold related to the camera resolution (e.g., $\theta \in (10^{-4},10^{-5})$).

### 3.2. Two-Step Algorithm for the Estimation of the Essential Matrix

The two-point algorithm, and consequently Algorithm 1, heavily rely upon the IMU measurements: hence, the quality of the obtained estimates strongly depends on the IMU measurement noise level. In order to make the estimation more robust, the two-step procedure shown in Algorithm 2 is considered.

**Algorithm 2:** Two-step algorithm
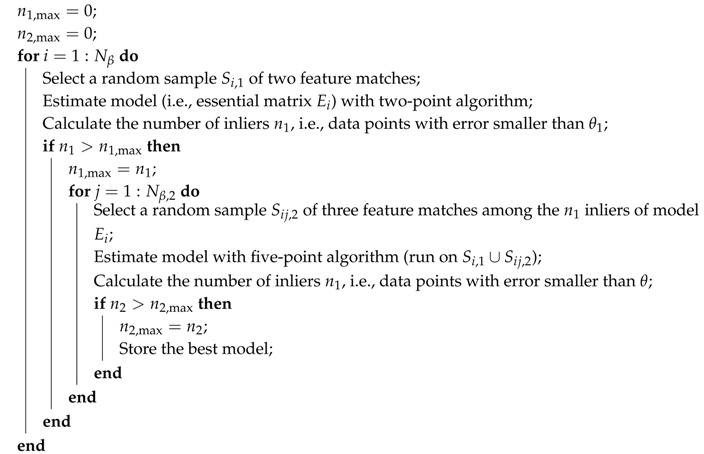


First, in the external loop, a RANSAC approach analogous to that of Algorithm 1 is used for determining a two-point estimate of the essential matrix (based on the procedure described in the previous subsection). Similarly to [44], in the external loop, the threshold for inlier detection is set to a larger value with respect to the desired one, e.g., $\theta_{1}=3\theta$ in the results of Section 4.

Then, in the internal loop, a RANSAC approach is used with a standard five-point algorithm in order to obtain a complete estimate of the essential matrix [60]: similarly to the locally optimized RANSAC [44], the RANSAC algorithm is executed considering only the inliers of the model determined in the external RANSAC loop. Furthermore, since two points are assumed to be already determined, in the internal loop, only three points can be randomly drawn. The number of loop iterations $N_{\beta ,2}$ is determined as in (8) by setting n=3. It is worth noticing that, since part of the incorrect feature matches are supposed to be removed during the external loop, the probability $\beta_{2}$ of correct feature matching in the internal loop is usually higher than that in the external loop, *β*. Nevertheless, unless specified differently, for simplicity in the following, $\beta_{2}=\beta$.

The internal loop is executed only when, in the external loop, a better two-point estimate is obtained (i.e., with a larger number of inliers with respect to the previous best two-point estimate): it is worth noticing that this happens only $\log N_{\beta }$ times [44], thus limiting the contribution of this step on the overall computational burden of the estimation procedure.

## 4. Results

The results shown in this section have been obtained by acquiring images and IMU measurements with an LG Google Nexus 5 (Android 4.4 KitKat) (LG, Seoul, South Korea), which is provided with a three-axis accelerometer, three-axis gyroscope, three-axis magnetometer, and 8 Mpixel camera. Nevertheless, similar results can be obtained by considering different devices and different operative systems.

### 4.1. Similarity Based Feature Matching

**Figure 3:**
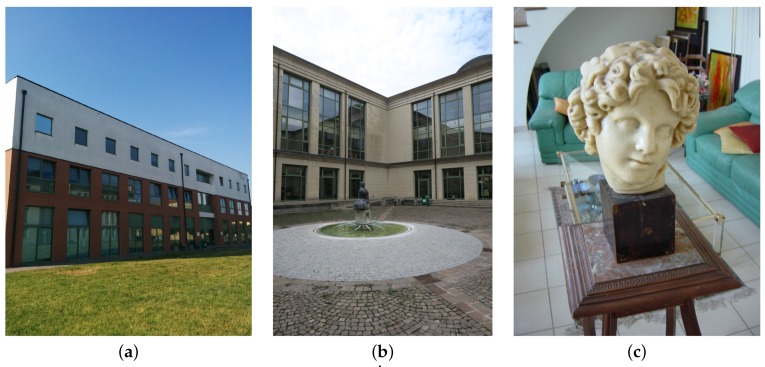
The considered case studies for the appearance based matching procedure presented in Section 2. (**a**) veterinary hospital and (**b**) Pentagono building of the University of Padova. (**c**) example of image from the website of [63]

The feature matching approach presented in Section 2 has been tested on three data-sets. Since this work aims at improving photogrammetric reconstruction for mobile mapping and surveying purposes (e.g., for monitoring human infrastructures), two considered case studies are university buildings, whereas, in order to make the test of interest in a more general context, the third case study is a small art object observed from very close positions:

- The first case study is a set of 11 images of the veterinary hospital of the University of Padova, Italy (Figure 3a).
- The second case study is a set of 17 images of the Pentagono building of the University of Padova, Italy (Figure 3b).
- The third case study is a set of images downloadable on the Internet from the website of [63] (Figure 3c). Since, in this case, IMU measurements are not available, they have been substituted with orientations computed after matching features in the images and adding to the computed orientation angles a Gaussian random noise with standard deviation 0.1 radiants (100 independent Monte Carlo simulations have been considered in order to provide statistically reliable results).

**Figure 4:**
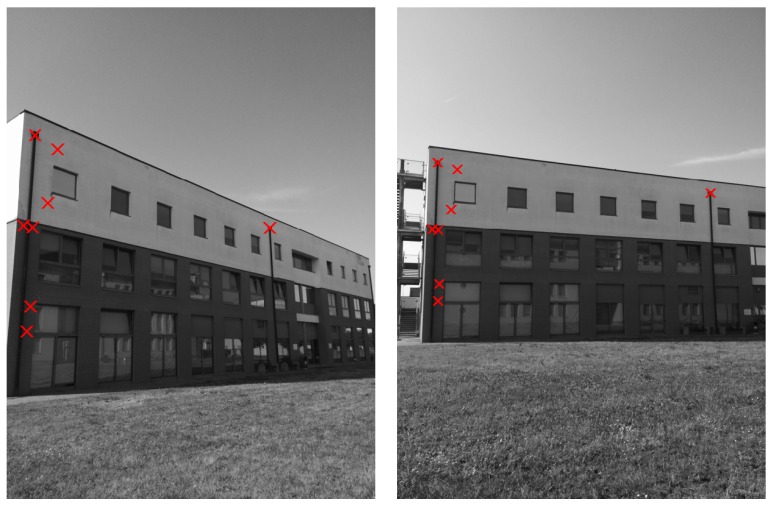
Example of feature points correctly matched by means of the SIFT descriptors.

**Figure 5:**
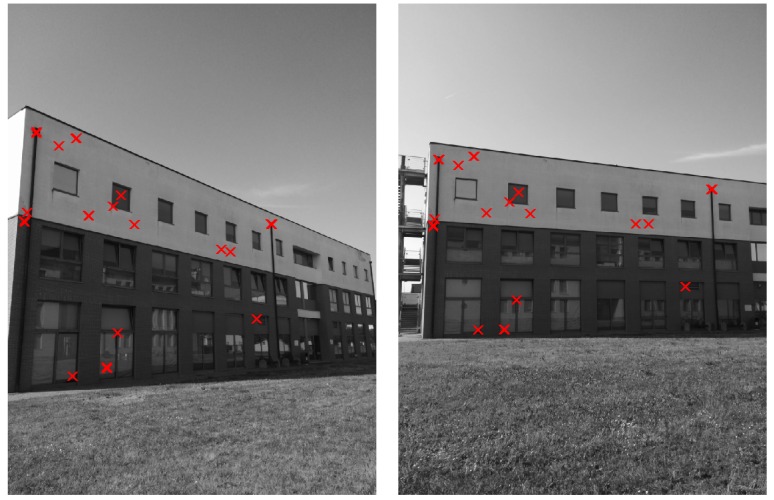
Example of feature points correctly matched by means of the technique proposed in Section 2 (N=1).

The method proposed in Section 2 is compared with the standard SIFT (feature locations and SIFT descriptors have been computed with VLFeat (version 0.9.20, A.Vedaldi and B.Fulkerson) [64] for both the considered methods). First, Figure 4 and Figure 5 show the correctly matched features with the two methods in a couple of images from the first data-set (h=1, the number of orientations to be used for comparison in the method of Section 2 has been set to one). Similar results can be obtained for each couple of images in the considered case studies.

**Figure 6:**
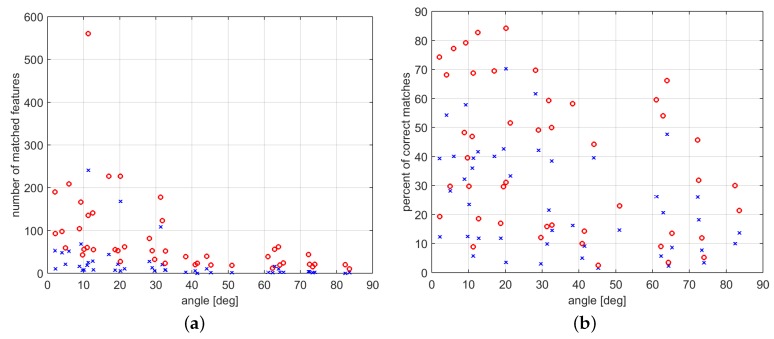
(**a**) number of correctly matched features and (**b**) percent of correctly matched features varying the angle between the camera poses. Comparison of number of matched features with standard SIFT (**blue** crosses), and with the method proposed in Section 2 (**red** circles) in the first case study.

**Figure 7:**
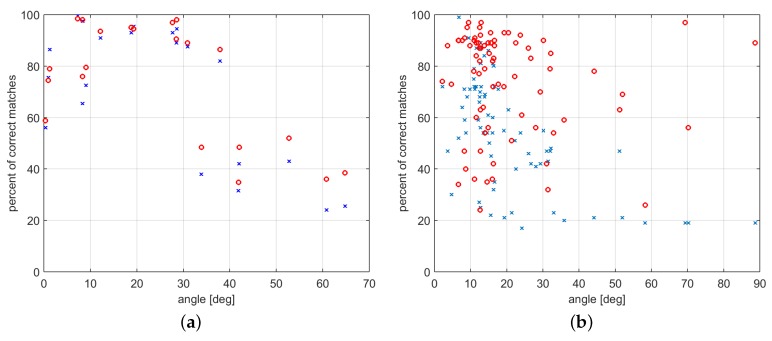
Percent of correctly matched features varying the angle between the camera poses. Comparison of number of matched features with standard SIFT (**blue** crosses), and with the method proposed in Section 2 (**red** circles) in the second (**a**), and third (**b**) case study, respectively.

**Figure 8:**
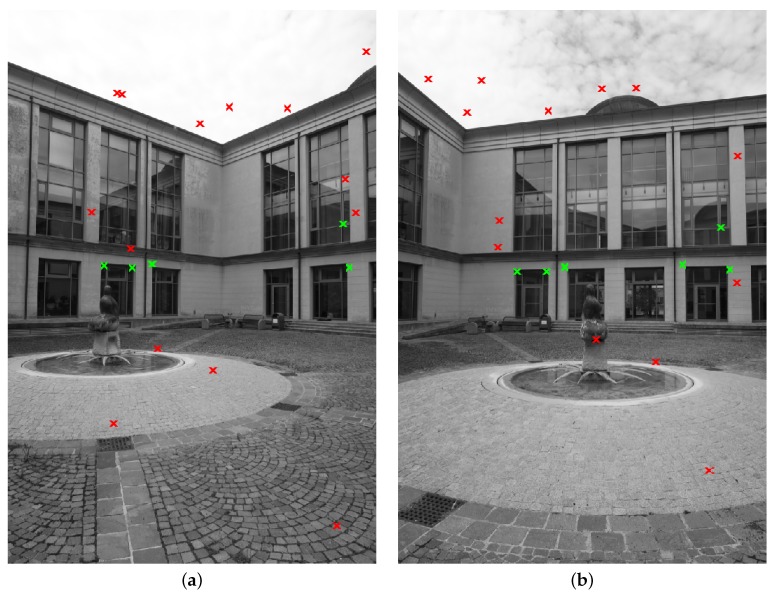
Incorrect matches (**red** and **green** *x*-marks) obtained with the method proposed in Section 2 in two images, (**a**) and (**b**), of the second case study. Errors related to repetitive shapes are shown as **green** *x*-marks.

The above consideration is experimentally proved by the results shown in Figure 6, Figure 7 and Figure 8. Let *R* be the rotation that (combined with a proper translation) relates two different camera poses, and let *ω* be the rotation angle associated with *R*. Then, Figure 6, Figure 7 and Figure 8 show the number of correctly matched features varying the value of *ω*, |ω|∈[0,π/2] in the three case studies (h=1, the number of orientations to be used for comparison in the method of Section 2 has been set to one).

**Figure 9:**
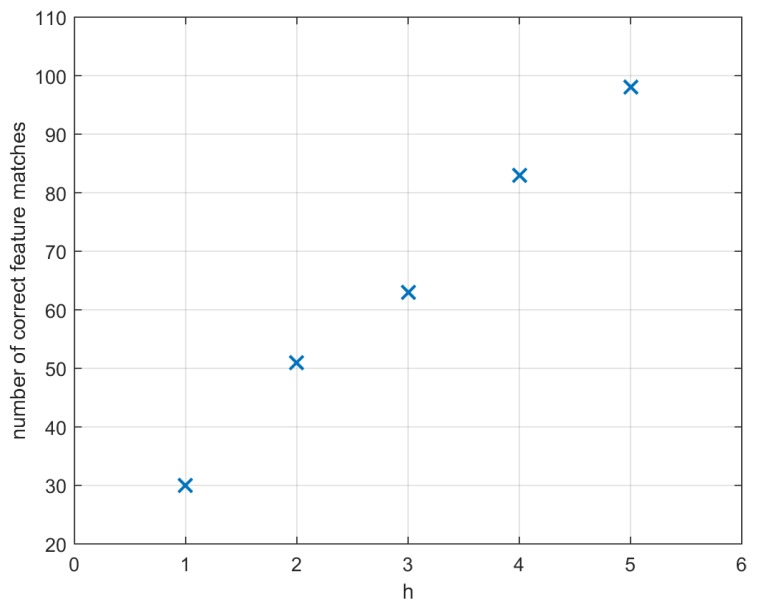
Number of correctly matched feature points by means of the technique proposed in Section 2 varying the number *h* of orientations to be compared: h={1,⋯,5}.

Furthermore, Figure 9 shows (in two images from the first case study, Figure 3a) the increase of the number of correct matches when considering more than just the closest orientation in the second image to that of the first one (h≥1).

### 4.2. Feature Matching with Geometry Constraints: Estimation of the Essential Matrix

In order to provide a statistically robust validation (not subject to errors related to other factors, e.g., inner camera parameters calibration) of the method presented in Section 3 for estimating the essential matrix, the method is first validated with a synthetic data-set in a Monte Carlo simulation:

- In each iteration of the Monte Carlo simulation, 50 feature points have been considered. Feature positions have been randomly sampled in a 10 m × 10 m × 3 m rectangular cuboid (uniform distribution).
- In each iteration of the Monte Carlo simulation, camera positions have been randomly sampled at a mean distance of 10m from the feature points. Camera orientations are obtained by summing a random Gaussian angle (zero-mean, standard deviation of π/18) to the direction pointing from the camera position to the cuboid center.

The method presented in Section 3 exploits the availability of IMU measurements in order to improve the essential matrix estimation: hence, the efficiency of the method changes depending on the measurement noise level of the IMU measurements. In order to obtain good results, IMU sensors are assumed to be properly calibrated [33,65,66], e.g., magnetometer systematic errors are (mostly) compensated by the calibration model. Then, let $\sigma_{0}$ be the standard deviation of the residual error after calibration of magnetometer of the LG Google Nexus 5.

Let $E_{21}$ be the real value of the essential matrix and ${\hat{E}}_{21}$ the estimated one. Then, ${\hat{E}}_{21}$ is considered as a good estimate of $E_{21}$ if ${∥E_{21}-{\hat{E}}_{21}∥}_{F}\leq \theta_{E}$, where ${∥\cdot ∥}_{F}$ stands for the Frobenius norm, and in the simulations of this section $\theta_{E}=0.35$.

**Figure 10:**
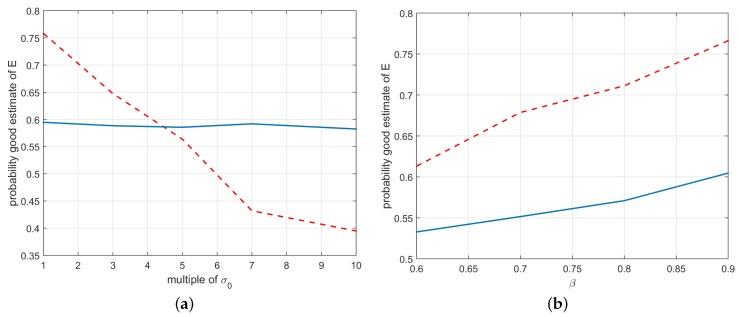
Probability of computing a good estimate of the essential matrix *E* varying the magnetometer measurement noise level (**a**) and *β* (**b**), respectively. Comparison of the results obtained with the standard five-point algorithm (**red** dashed line), and the two-point algorithm of Section 3.1 (**blue** solid line).

Figure 10a shows the estimated probability of obtaining a good estimate of the essential matrix while varying measurement noise level of the magnetometer, i.e., varying the standard deviation $\sigma_{m}$ of the magnetometer noise ($\sigma_{m}=\sigma_{0}$, $2\sigma_{0}$, $3\sigma_{0}$, ⋯), whereas *β* is fixed to a constant value (β=0.85).

The figure compares the results obtained with the standard five-point algorithm (red dashed line), and the two-point algorithm proposed in Section 3.1 (blue solid line). Since the standard five-point algorithm does not exploit IMU measurements, its results are obviously constant with respect to the magnetometer measurement noise level. Figure 10b shows the estimated probability of obtaining a good estimate of the essential matrix while varying the correct match probability *β* (whereas the magnetometer measurement noise level is fixed to a constant value, $\sigma_{m}=\sigma_{0}$)). Results reported in Figure 10 are the average of 100 independent Monte Carlo simulations.

**Figure 11:**
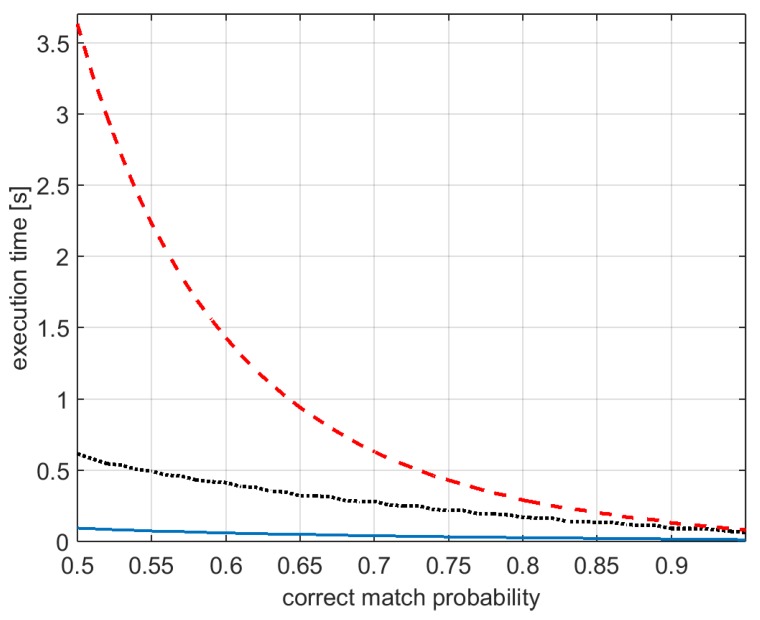
Computational time for obtaining estimates of the essential matrix varying *β*. Comparison of the results obtained with the standard five-point algorithm (**red** dashed line), Algorithm 1 of Section 3.1 (**blue** solid line), and the two-step algorithm (Algorithm 2) of Section 3.2 (**black** dotted line).

Figure 11 shows an estimate of the execution time for computing essential matrix varying the correct match probability *β* (magnetometer measurement noise level is fixed to a constant value, $\sigma_{m}=\sigma_{0}$). The figure compares the standard five-point algorithm (red dashed line), the two-point (solid blue line) of Section 3.1 and the two-step algorithm (dotted black line) of Section 3.2. Notice that the execution times actually depends on the computational power of the device running the algorithms: results shown here are based on results obtained with Matlab (version R2015a, MathWorks, Natick, MA, USA)^®^ on a desktop with CPU Intel (Santa Clara, CA, USA) ^®^@2.20GHz.

Table 1 shows the accuracy (estimated as the average result of 100 independent Monte Carlo simulations, in the synthetic case study presented at the beginning of this subsection) of the considered methods varying the magnetometer measurement noise level. It is worth noticing that the results in different rows of the table have been obtained in different conditions (e.g., different independent Monte Carlo simulations): hence, only the numerical results in the same row should be compared. The best result in each row is reported in bold.

**Figure 12:**
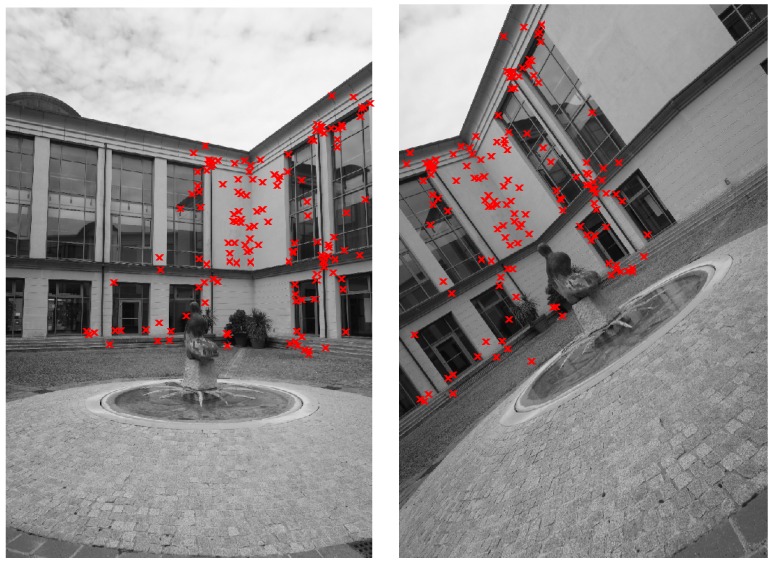
Example of feature points matched by taking into consideration geometry constraints (second case study).

Finally, Figure 12 shows the feature points matched by taking into consideration geometry constraints (outlier rejection determined by means of Equation (9)) in two images of the second case study of Section 4.1. Algorithm 2 and the standard five-point algorithms have been executed 50 times in order to evaluate the stability of the obtained results. Setting the RANSAC simulation parameters in such a way to obtain similar stability results (mean standard deviation of 0.07 on the values of the essential matrix entries), the following average computational times have been measured: 4.9 s for the standard five-point algorithm and 1.5 s for Algorithm 2.

## 5. Discussion

As shown in Figure 6, Figure 7 and Figure 8 (and in the example of Figure 5), the method proposed in Section 2 allows for significantly increasing the number of correctly matched feature points between two images with respect to the SIFT. Furthermore, interestingly, this property holds for a wide range of values of the relative angle *ω* between the two camera views (Figure 6, Figure 7 and Figure 8 for 0≤|ω|≤π/2). Depending on the considered case study, the fraction between the average number of correctly matched features with the proposed method and with standard SIFT ranges from two to ten (using h=1).

The increase of correct feature matches can be further improved by increasing the number of closest orientations *h* considered in the matching procedure of Section 2 (Figure 9). However, the computational complexity of the proposed procedure increases linearly with respect to *h*: for h=1, the number of comparisons involved in the matching procedure is approximately *N* times smaller than in the ASIFT case (Section 2), whereas for large values of *h*, the computational complexity of the proposed method becomes similar to that of the ASIFT, hence reducing the difference between the two. Furthermore, despite the increase of correct feature matches appearing linear with *h* in Figure 9, this does not hold for large values of *h*: the increase of correct feature matches becomes smaller as the value of *h* becomes larger. These considerations motivate the use of a small value of *h* in practical applications where computational time constraints are relevant, e.g., h≈1.

Algorithm 1 proposed in Section 3.1 exploits the IMU measurements in order to reduce the degrees of freedom to be estimated in the essential matrix from five to two. When the magnetometer measurements are reliable, the need of only two feature correspondences for executing the estimation procedure allows for significantly increasing the probability of computing a good estimate of the essential matrix (Figure 10b) while dramatically reducing the computational burden of the algorithm (Figure 11). However, the probability of computing a good estimate of the essential matrix (and hence of properly detecting wrong feature matches and estimating the relative camera poses) significantly decreases when the magnetometer measurement noise level becomes higher (Figure 10a), making the method inefficient for large noise levels.

This motivated the introduction of a two-step algorithm, Algorithm 2, for making the results of the procedure less influenced by the magnetometer measurement noise level: as shown in Table 1, the two-step algorithm is much less sensitive to the magnetometer measurement noise level with respect to Algorithm 1. However, its estimate is obviously still influenced by the magnetometer measurement noise level: when such noise level is moderate, Algorithm 2 performs better than the five-points algorithm, whereas when it is large the standard five-points algorithm becomes more accurate. In practice, the introduction of magnetometer measurements in the essential matrix estimation procedure has the effect of increasing the robustness for low–moderate levels of the magnetometer measurement noise, while it reduces the estimation accuracy for high levels of the measurement noise.

As shown in Figure 11, Algorithm 2 is computationally less effective in terms of computational time than Algorithm 1; however, it is definitely faster than the standard five-point algorithm for not so high values of the correct match probability *β*.

Figure 12 shows the results of the proposed Algorithm 2 for what concerns the rejection of incorrect feature matches in two images from the case study of Figure 3b. In this example, Algorithm 2 obtained similar results to the standard five-point algorithm in terms of accuracy of the estimation of the essential matrix, while reducing the computational burden by a factor of three, approximately (1.5 s and 4.9 s, respectively).

## 6. Conclusions

In this paper, the process of feature matching and camera pose estimation (which is a step of fundamental importance in image based 3D reconstructions) has been reviewed by taking into account the availability of IMU measurements when using recent mobile devices.

First, a new method for increasing the number of appearance based correct feature matches between two images has been presented: the proposed method can be considered as a revision of the ASIFT method when camera orientation information (measured by the device sensors) is available. The results in the considered case studies showed that the presented method allows for significantly increasing the number of appearance based correct feature matches with respect to the SIFT, while reducing the computational burden of the ASIFT.

Then, IMU measurements have been used in order to improve the process of feature matching with geometry constraints: the proposed two-point algorithm allows for quickly obtaining estimates of the essential matrix; however, it is quite sensitive to the magnetometer measurement noise level, becoming inefficient for when such noise becomes larger. The two-step algorithm proposed in Section 3.2 allows for reducing the sensitivity of the estimation procedure with respect to the magnetometer measurement noise level, while still being typically faster than the standard five-point algorithm. Nevertheless, since both Algorithms 1 and 2 make use of the IMU measurements, their convenience with respect to the standard five-point algorithm reduces as the IMU noise level increases: this makes an accurate IMU sensor calibration of fundamental importance in order to make the proposed procedures more effective.

## Figures and Tables

**Table 1:** Accuracy of the algorithms for estimating the essential matrix varying the magnetometer measurement noise.

Noise Level $\sigma_{m}$	Five-Point Algorithm	Two-Point Algorithm	Two-Step Algorithm
$\sigma_{0}$	0.33	**0.22**	0.26
$2\sigma_{0}$	0.18	0.21	**0.15**
$3\sigma_{0}$	**0.15**	0.41	0.18
